# Survival of contagious ovine digital dermatitis (CODD)-associated treponemes on disposable gloves after handling CODD-affected feet

**DOI:** 10.1136/vr.104228

**Published:** 2017-07-22

**Authors:** J. W. Angell, S. R. Clegg, D. H. Grove-White, R. W. Blowey, S. D. Carter, J. S. Duncan, N. J. Evans

**Affiliations:** 1 University of Liverpool, Brownlow Hill,, Liverpool, UK; 2 Wood Veterinary Group, Gloucester, UK

## Context

Contagious ovine digital dermatitis (CODD) is related to bovine digital dermatitis, both being strongly associated with the pathogenic treponemes: *Treponema medium*, *Treponema phagedenis* and *Treponema pedis*. Other *Treponema* species-associated diseases with distinct clinical presentations have also been identified in goats and wild elk. In sheep, other causes of infectious lameness are also common, with footrot (caused by *Dichelobacter nodosus*) being shown to be strongly associated with CODD.

CODD is now common in the UK with approximately 50 per cent of farms affected. A recent study isolated the treponemes from knives used for hoof trimming infected cattle and sheep, and while contact between animals is considered the most likely route of transmission, this has raised the issue that manual transmission by people could be possible. 

When inspecting or treating infected feet, wearing gloves is a reasonable personal hygiene and biosecurity measure, therefore this study aimed to investigate the potential to transmit CODD-associated *Treponema* species via gloves used when handling clinically affected animals.

## Main conclusion

Handling sheep may lead to the transmission of pathogenic *Treponema *species between sheep with and without CODD and possibly between farms. *Treponema* species associated with CODD lesions can survive on gloves used to handle affected feet for up to three days in air and may represent a biosecurity risk.

## Approach

Three related experiments were carried out. Experiment 1 was a case-control study which compared the presence of CODD-associated *Treponema* species on gloves following the handling of clinically affected and clinically unaffected feet. Clinical cases (n=23) were identified as sheep with clinically active CODD. Control sheep, matched by farm and grazing group (n=30) were those with clinically normal feet. All the feet were handled as part of routine examination using sterile disposable gloves. The gloves were then swabbed to detect the presence of treponemes immediately after handling. The association between CODD-associated treponemes and *D nodosus* and *Fusobacterium necrophorum* was also investigated. 

Experiment 2 was a longitudinal study which examined the duration of survival of the CODD-associated *Treponema* species in air. Swabs were taken on five sequential days from one of five gloves used to handle the same foot with CODD (n=12) to determine the viability of the *Treponema* species over time. 

For experiment 3, gloves from both experiments 1 and 2 were used to determine the viability of *Treponema *species following cleaning with various solutions. Detection methods included culture and isolation techniques together with DNA detection by PCR.

## Results

Experiment 1: no CODD-associated treponemes were detected from the gloves used to handle the control sheep. CODD-associated treponemes were detected by culture from 91 per cent (95 per cent confidence interval [CI] 69 to 98 per cent) of the swabs taken from gloves used to handle the CODD cases and from 100 per cent of cases tested by PCR. Growth of treponemes occurred in inoculates from 24 of the samples taken, confirming that the bacteria were viable. For those gloves used to handle CODD-affected feet (n=23), there was no association between the presence of CODD-associated *Treponema* species and either *D nodosus* or *F necrophorum*, individually or in combination (P>0.1).

Experiment 2: for *T medium*, bacteria were cultured and detected using PCR from 67 per cent of gloves (95 per cent CI 33 to 89 per cent) and were viable for up to one day. For *T phagedenis*, bacteria were cultured from 75 per cent (95 per cent CI 43 to 95 per cent) of gloves and detected by PCR from 83 per cent (95 per cent CI 46 to 97 per cent). For *T pedis*, bacteria were cultured and detected by PCR from 67 per cent of gloves (95 per cent [CI] 33 to 89 per cent). For both *T phagedenis* and *T pedis*, the treponemes were viable on gloves for up to three days. For all the sheep where treponemes were detected from the gloves by PCR, the treponemes remained detectable for all five days. 

Experiment 3: CODD-associated treponemes were detected by culture and PCR from all 10 gloves following cleaning by washing with cold and warm water. However, none were detected by culture following cleaning with water and hand soap, a 1 per cent concentration of Virkon solution, a 1:90 dilution of FAM or 70 per cent ethanol, although CODD-associated treponemes were detectable by PCR on one of the gloves cleaned with hand soap and one with Virkon. 

## Interpretation

Treponemes are considered to be anaerobic, however, the survival of treponemes on gloves for two to three days in air suggests that these bacteria may not be strict anaerobes and are able to tolerate oxygen for short periods. Careful disposal of gloves is necessary to prevent inadvertent infection through contact with gloves, and it is possible that being able to survive for short periods in aerobic conditions may increase the chances of survival on the farm. It also raises the possibility of a risk of transmission between farms by contaminated material being transferred on personnel and equipment and reiterates the need for rigorous farm biosecurity policies.

## Significance of findings

CODD-associated treponemes are present (and can survive) on the gloves of personnel working with CODD-affected sheep feet. A preventive strategy to limit this potential route of transmission could involve changing gloves, or at the very least cleaning gloves with an appropriate disinfectant solution, following the handling of affected sheep.

